# Third trimester intrauterine fetal death: proposal for the assessment of the chronology of umbilical cord and placental thrombosis

**DOI:** 10.1007/s00414-022-02784-3

**Published:** 2022-02-11

**Authors:** Maria Paola Bonasoni, Barbara Muciaccia, Caterina B. Pelligra, Matteo Goldoni, Rossana Cecchi

**Affiliations:** 1 Pathology Unit, Azienda Unità Sanitaria Locale, IRCCS, Reggio Emilia, Italy; 2 Department of Anatomical, Histological, Legal Medical and Orthopaedic Sciences, Faculty of Medicine and Pharmacology, Sapienza University of Rome, Rome, Italy; 3 Department of Medicine and Surgery, Laboratory of Legal Medicine, University of Parma, Parma, Italy; 4 Department of Medicine and Surgery, Medical Statistics, University of Parma, Parma, Italy

**Keywords:** TT-IUFD, Fetal thrombosis, Timing of thrombus, Medical litigation, Forensic obstetrics

## Abstract

The timing of umbilical cord and placental thrombosis in the third trimester intrauterine fetal death (TT-IUFD) may be fundamental for medico-legal purposes, when it undergoes medical litigation due to the absence of risk factors. Authors apply to human TT-IUFD cases a protocol, which includes histochemistry and immunohistochemistry (IHC) for the assessment of thrombi’s chronology. A total of 35 thrombi of umbilical cord and/or placenta were assessed: 2 in umbilical artery, 6 in umbilical vein, 15 in insertion, 10 in chorionic vessels, 1 in fetal renal vein, 1 in fetal brachiocephalic vein. Thrombi’s features were evaluated with hematoxylin–eosin, Picro-Mallory, Von Kossa, Perls, and immunohistochemistry for CD15, CD68, CD31, CD61, and Smooth Muscle Actin. The estimation of the age of the thrombi was established by applying neutrophils/macrophages ratio taking into consideration, according to literature, the presence of hemosiderophagi, calcium deposition, and angiogenesis. To estimate an approximate age of fresh thrombi (< 1 day), a non-linear regression model was tested. Results were compared to maternal risk factors, fetal time of death estimated at autopsy, mechanism, and cause of death. Our study confirms that the maternal risk factors for fetal intrauterine death and the pathologies of the cord, followed by those of the placental parenchyma, are the conditions that are most frequently associated with the presence of thrombi. Results obtained with histological stainings document that the neutrophile/macrophage ratio is a useful tool for determining placental thrombi’s age. Age estimation of thrombi on the first day is very challenging; therefore, the study presented suggests the N/M ratio as a parameter to be used, together with others, i.e., hemosiderophagi, calcium deposition, and angiogenesis, for thrombi’s age determination, and hypothesizes that its usefulness regards particularly the first days when all other parameters are negative.

**Supplementary Information:**

The online version contains supplementary material available at 10.1007/s00414-022-02784-3.

## Introduction

According to the World Health Organization (WHO), third trimester intrauterine fetal death (TT-IUFD) is considered the birth of a child showing no signs of life that occurs from the 28th week of pregnancy onwards, whereas many differences can be found between different states (http://www.who.int/maternal_child_adolescent/epidemiology/stillbirth/en/). In Italy, specifically in Emilia Romagna Region, TT-IUFD is defined as the loss of a fetus at a gestational age of at least 22 weeks. When the gestational age is not known or not calculable, a stillborn with a birth weight of 500 gr or a length of 25 cm is classified as TT-IUFD [1].

The difficulties encountered in the diagnosis and international classification of intrauterine death are influenced by the multiplicity of possible causes and factors related to fetal death [2].

According to the Lancet Ending Preventable Stillbirths study group, late gestation stillbirth rates vary across high-income countries from 1.3 to 8.8/1000 births. However, these results may further be reduced with correct analysis of risk factors (obesity, advanced maternal age, in vitro fertilization), access to antenatal healthcare, accurate monitoring during pregnancy, improved data from stillbirth autopsies performed by a trained perinatal pathologist, and optimizing bereavement care [3].

Regardless of definitions and statistics, there are several reasons why it is important to deal with TT-IUFD. Stillbirth is still an unknown phenomenon, not allowing appropriate interventions to prevent and reduce its frequency. Problems related to the frequent lack of consent to autopsy, or to the absence of fetal and/or placenta examination, reduce the possibility to identify the cause of death.

Understanding the reasons for the event is important either for the relationship between doctor and patient, or for the care planning of future pregnancies. This may ensure adequate support for mothers and family members [4].

Furthermore, the lack of information on TT-IUFD leads to the high frequency of medico-legal litigation, which, besides representing a very long-lasting drama for family members and for the doctor, raises overall healthcare costs. Currently, also in Italy, obstetric practice has an increasing number of medico-legal implications. Moreover, the reduced number of births results in a great expectation towards pregnancy and its outcome. The increased average age of pregnant women raises risk factors, which can inevitably lead to an enhancement in TT-IUFD incidence.

Maternal risk factors for TT-IUFD are diabetes, obesity, hypertension, and thrombophilia. A high number of pregnancies resulting in a TT-IUFD do not present maternal risk factors and lead therefore to medico-legal disputes. In these cases, it is of paramount importance to dispose of useful elements to diagnose the fetus-placental suffering and its time of onset, in order to verify if there has been a delay in the gynecological intervention. A useful aid in this sense is given by the estimated age of any present thrombi that, together with other data, help timing the fetus-placental suffering.

Placental anomalies are the most common cause of TT-IUFD (more than 60%); however, a good percentage (about 25%) still occurs due to unknown causes [5]. From a review made by Ptaceck et al. [6], more than 30% of TT-IUFD are due to placental abnormalities such as abruption, infarction, chorioamnionitis, villous dysmaturity, HEV, insufficiency, perivillous fibrin deposits, placental chorangioma, retroplacental hematoma, feto-maternal hemorrhage, placenta praevia, cord accident, and hydrops. Nevertheless, the precise role of placental lesions in fetal mortality remains uncertain, and the determination of causality in a single case is difficult especially when no maternal risk factors are known. This is due to differences in diagnostic criteria and in classification of clinical information.

Vascular lesions, especially thrombosis, are the second most common cause of fetal damage in the last weeks of pregnancy, including death [7]. The umbilical cord thrombosis has an incidence from 1 to 10 in the stillbirths [8]. It is usually linked to villous dysmaturity or cord anatomical anomalies (hyper- or hypocoiling, restriction, anomalous insertions in the placenta), as well as compression, torsion, or entanglement around fetal parts and true knots.

An organized thrombus may indicate a long-lasting fetal suffering, while fresh thrombi, dated less than few hours, usually may indicate an acute fetal suffering. This may lead to different medico-legal scenarios.

In literature, there are very few studies concerning the timing of thrombi. Irniger et al. [9] and Fineschi et al. [10] propose classifications of the histological age of thromboses and embolisms. Both studies refer, besides erythrocytes, platelets, and fibrin, an immediate presence of white blood cells up to the third day, when white blood cells become pyknotic and monocyte increase in number with enlarged nuclei. In the Irniger’s study starting from day 4th (phase III: 4th–20th day), the first capillaries are seen together with fibroblasts, mesenchymal cells, and hemosiderin-accumulating histiocytes. In Fineschi’s study, calcium, as precipitates with von Kossa stain, is seen within the first week, while fibroblasts’ penetration is seen from the 2nd week. Calcium precipitation is due to the action of the phosphatases released following the cell lysis that precipitates the calcium salts.

A more detailed timing of thrombi formation and characterization is available on mouse model by Nosaka et al. [11, 12].

Unfortunately, until now, studies on human thrombi never focused on a reliable marker of chronology, and all information refer to large timeframes of days or weeks. In medico-legal litigation cases, the timing of events can be of crucial importance, and, for what concern thrombi, especially the first hours or days [13].

Starting from the general agreement that white cells are immediately predominant in very early thrombi, and with time the number of monocytes increases, in the present study, the ratio between neutrophiles and macrophages is investigated and related to the timing of thrombi of placental and umbilical cord human samples. Besides, according to the main characteristics of the thrombogenesis and its evolution, the presence of fibroblasts and neovascularization as well as hemosiderin and calcium precipitates staining were evaluated.

## Material and method

From 2014 to 2017, a total of 320 fetal autopsies were performed at the Pathology Unit of Reggio Emilia Hospital. Thirty-six cases were TT-IUFD. All the autopsies were performed by the same staff and in accordance with international protocols, including the macroscopic evaluation of the placenta and the fetus, and the histological evaluation. All autopsies of fetuses delivered after voluntary termination of pregnancy and fetuses with anatomical malformations or genetic alterations were excluded from the study.

Placental histology included sections from cord, insertion of the cord into the chorionic plate, chorionic vessels, and the parenchyma.

In 19 cases, one or more umbilical cord and/or placental thrombi were found and were included in this study. The cause of death were as follows: cord turns around the neck or other parts of the fetal body (4), placental dysmaturity (5), corionamnionitis, necrotizing funisitis and amnionitis (4), cord compression (2), placenta infarction (2), maternal–fetal hemorrhage (1), cord knot (1).

For all cases, umbilical cord anomalies, placental dysmaturity, cord turns around the neck or other parts of the fetal body, and maternal risk factors were considered.

A total of 35 thrombi were assessed: 2 in umbilical artery, 6 in umbilical vein, 15 in insertion, 10 in chorionic vessels, 1 in fetal renal vein, 1 in fetal brachiocephalic vein.

All thrombus samples were formalin-fixed and paraffin-embedded.

Thrombus features were evaluated with hematoxylin–eosin staining and Picro-Mallory staining for fibrin. Von Kossa was performed to confirm the presence of calcium, and Perl’s staining was applied to detect hemosiderin + cells. Furthermore, for immunohistochemical evaluation of cells type, polyclonal antibodies were used: CD15 for neutrophils (Clone MM1, Ventana Group, Milan, Italy), CD68 PGM1 for macrophages (Clone PGM1, DBS, Pleasanton, CA, USA), CD31 for endothelial cells (Clone JC70, Cell Marque, Rocklin, CA, USA), and smooth muscle actin antibody for myofibroblasts (Clone 1A4, Cell Marque, Rocklin, CA, USA).

Each thrombus was classified as occluding, sub-occluding, or mural. Picro-Mallory staining for fibrin and immunohistochemistry for CD61 for platelets (Clone 2F2, Cell Marque, Rocklin, CA, USA) were used to discriminate between organized thrombus and post-mortem clots.

The number of CD15 + and CD68 + cells was assessed in 5 fields at 60 HPF, and the N/M ratio was statistically evaluated for thrombus age timing. For each thrombus neovessel organization and the presence of hemosiderin + cells, myofibroblasts, platelets, and von Kossa positive calcium deposition was detected.

### Statistical method

In order to verify if the N/M ratio can be reliable for thrombi’s age determination, the Levenberg Marquardt algorithm was used for statistical analyses.

## Results

In the 19 autopsies performed, associations between the thrombus and the maternal-placental risk factors were always found. Particularly, maternal pathologies, anatomical anomalies of placenta, and cord were documented as follows:

- umbilical cord anomalies (14 cases): 8 cases showed abnormal insertion of the cord into the placenta, 8 documented abnormalities such as knots, hyper-, or hypocoiling; in two cases, more anomalies were co-existent;
- placental dysmaturity (11 cases);
- cord turns around the neck or other parts of the fetal body (4 cases);
- maternal risk factors like diabetes, obesity, preeclampsia, thyroid disease, or Leiden factor V in heterozygosity (8 cases);
- no maternal risk factors (11 cases).

Of the 35 thrombi found, 15 were localized in the insertion of the funiculus on the placenta, 10 in the chorionic vessels, 6 in the umbilical vein, and 2 in the umbilical artery. Two other thrombi were found in the renal vein and brachiocephalic trunk of a fetus, respectively. Only 1 thrombus was occluding (in the renal vein), while all other were non-occluding.

**Fig. 1:**
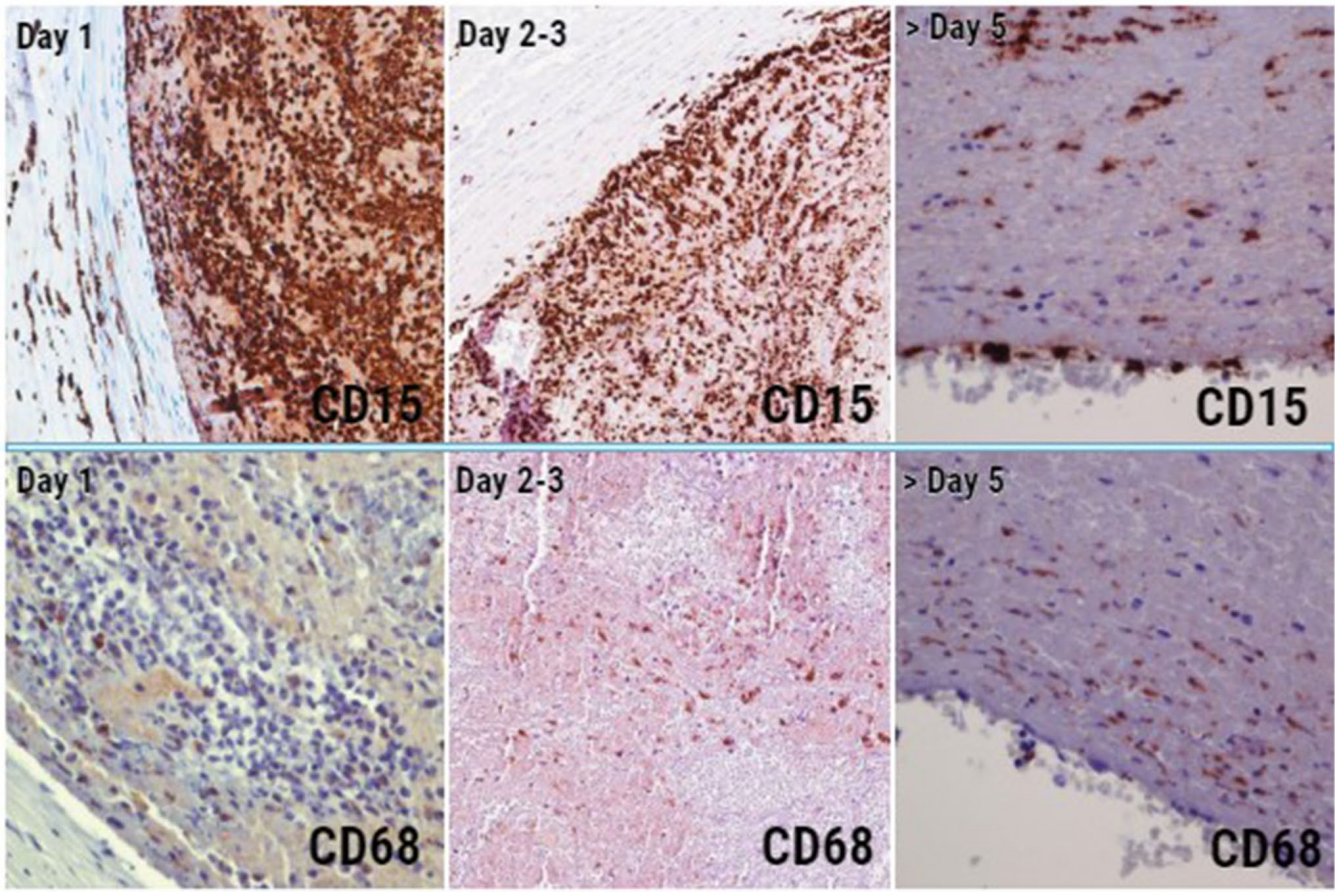
Comparison between neutrophils (CD15 +) and macrophages (CD68 +) in different timeframes thrombi. Day 1, chorionic vessel (CD15 4HPF, CD68 10 HPF); day 2–3, cord vein (CD15 4HPF, CD68 10 HPF); day 5, cord insertion (CD15 10HPF, CD68 10 HPF). Over time, the number of neutrophils decreases and that of macrophages increases

Morphological analysis of thrombus samples documented that the increase of the number of macrophages was related to a decrease in neutrophils (Fig. 1). This suggested to assume the strongest presence of neutrophils as a marker of very early thrombogenesis, and the decrease of N/M ratio as a potential marker of thrombus evolution.

**Fig. 2:**
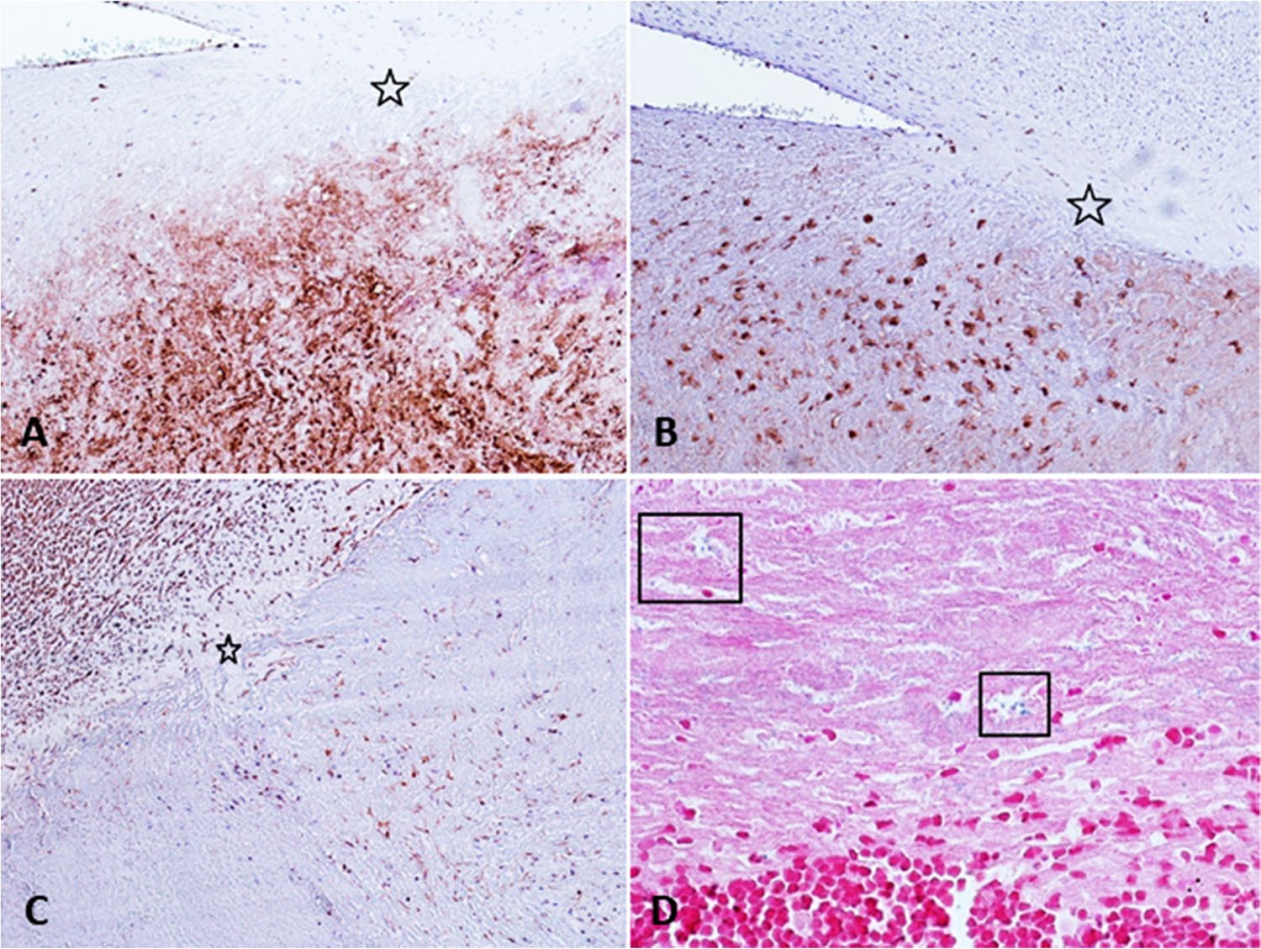
IHC with anti-CD15 (**A**), anti-CD68 (**B**), anti-SMA (**C**), and Perl’s staining (**D**) in a thrombus classified as > 7 days old. Stars in **A**, **B**, **C**: endothelial budding of the thrombus to the vessel wall. Black frames in **D**: iron deposition Perl’s + within macrophages

Histological analysis performed on the 35 thrombi respectively revealed the Perls staining positivity in 12 samples while von Kossa staining was detected in 6 samples. Regarding immunohistochemistry, CD31 positive cells were detected in 7 out of the 35 thrombus samples, while smooth muscle positive cells were observed in 5 (Fig. 2).

As reported in the Online Resource 1, the N/M ratio found varied from 28 to 0.2. All samples showed positivity for CD61 staining (cluster or scattered distribution of platelets) and Picro-Mallory (fibrin diffuse or organized in strands) proving to be thrombi and not post-mortem clots. No relation between N/M ratio and the type of distribution of platelets and fibrin was found. In the N/M ratio range of 28.0 < 1.4, all samples studied were negative for hemosiderin + cells (Perls staining), neovascularization (CD31), calcium deposition (von Kossa), and myofibroblasts (smooth muscle actin). At N/M ratio, 1.4 hemosiderin + cells and calcium deposition started to be found in some samples, while neovascularization at N/M ratio of 1.1, and myofibroblasts penetration at N/M ratio of 0.6. These results allowed to hypothesize a timing of thrombogenesis in which the decrease of N/M ratio correspond to an increase of thrombus age.

According to Irninger, the presence of hemosiderin accumulated in histiocytes, first capillaries, and fibroblasts appear at phase III (4th–20th day), and calcium deposition, according to Fineschi et al., is found in the first week. In our cases, the appearance of these features started at N/M ratio = 1.4, and at N/M ratio 0.2 ≤ 0.6, all features were present in some cases (angiogenesis, hemosiderophagi, calcium deposition, myofibroblasts penetration). It can be therefore assumed that the range of N/M ratios 1.4–0.2 falls into the Irninger phase III. Starting from this assumption, the results were disposed in a decrescent order of N/M ratio and compared to the experimental results obtained by Nosaka et al. in a mouse model [11].

In the mouse model, the N/M ratio of 1.1 corresponded statistically to a thrombus age of 5 days and values from 1.4 to 0.2 were found only in thrombi more than 5 days old. These results agree with those of our study in which only the N/M ratios within 1.4 and 0.2 matched, as seen before, with the presence of parameters well known to belong to thrombus older than 4 days.

**Fig. 3:**
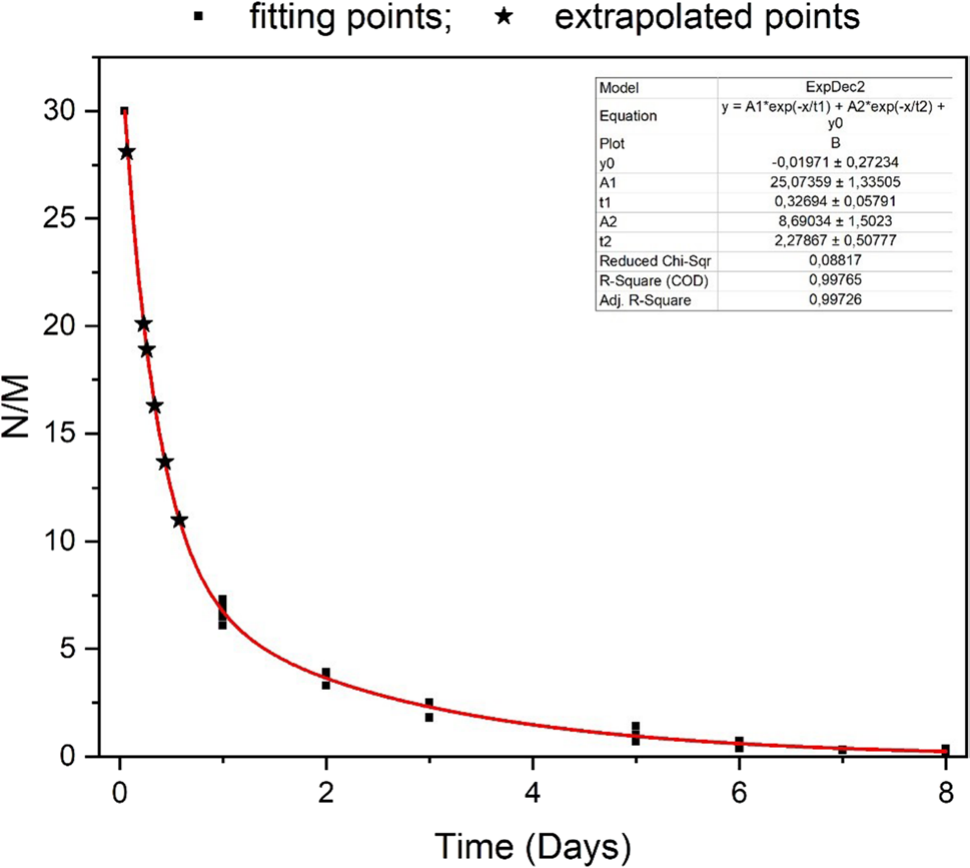
Considering the highest N/M ratio of 28 as the nearest to the very first hour of thrombus formation, and comparing the other ratios between them, the Levenberg Marquardt algorithm allows to document many fitting points between the mouse and the human model, and to hypothesize the N/M ratios of the first 24 h

Keeping in mind that in experimental models, the intravenous thrombus development is stasis-induced, and endothelial injury and blood hypercoagulability are not present; the hypothesis that N/M ratios found in mouse model could possibly match those in human model verified applying the bi-exponential decay non-linear model called Levenberg Marquardt algorithm, and results of both studies—mouse and human model—were considered together. Considering the highest N/M ratio of 28 as the nearest to the very first hour of thrombus formation, and comparing the other ratios between them, the Levenberg Marquardt algorithm permitted to document many fitting points between both studies, and to extrapolate the N/M ratios of the first 24 h (Fig. 3 and Table 1).

**Table 1:** Extrapolation of six approximate timeframes for fresh thrombi through the application of the bi-exponential decay non-linear model

Timeframe of thrombi (hours)	N/M ratio
1–2	28.1
5–6	20.1
6	18.9
8	16.3
11	13.7
13	11
24	6.8 ± 1.1

According to the obtained results, the matching of thrombus localization and timing shows very early thrombi (1 day old) mostly in the cord (6 thrombi), followed by the insertion (3 thrombi) and chorionic vessels (3 thrombi). Early thrombi (2–3 days old) were found in the insertion (3 thrombi), followed by cord (2 thrombi) and chorionic vessels (1 thrombi). Four- to six-day-old thrombi were documented in chorionic vessels (2 thrombi) and in the insertion (1 thrombus), while thrombi aged more than 7 days were often found in the insertion (8 thrombi) and less in the chorionic vessels (4 thrombi). In one case, two thrombi were found, one aged 4–6 days in the brachiocephalic artery, and one > 7 days old in the renal vein (Online Resource 1).

## Discussion

In all 19 cases studied, autopsy revealed risk factors connected with fetus or placenta and confirmed that TT-IUFD is related to villous dysmaturity or cord anatomical anomalies (hyper- or hypocoiling, restriction, anomalous insertions in the placenta), as well as compression, torsion, or entanglement around fetal parts and true knots.

The cause of death was identified in cord turns around the neck or other parts of the fetal body, placental dysmaturity, chorioamnionitis, necrotizing funisitis and amnionitis, cord compression, placenta infarction, maternal–fetal hemorrhage, and cord knot.

To be outlined, the lack of maternal risk factors in 11 out of 19 autopsies. These might have led to medico-legal disputes making it in some cases imperative to chronologically define the onset of the fetus-placental suffering in order to verify if there has been a delay in the gynecological intervention.

In our study, it had not been possible to document any correlation between the formation, location, and chronology of the thrombi and the cause of death, or the fetal and maternal risk factors.

Most of the thrombi were localized in the insertion of the cord in the placenta (15 out of 35); 8 of them were more than 7 days old. This is probably due to the turbulence of the blood flow in this district, which may favor a hemodynamic distress that leads to a wall shear stress, which promotes endothelial activation with shape change, and secretion of substances that induces vasoconstriction, coagulation, and platelet aggregation [14, 15].

Very early (1 day old) and early thrombi (2–3 days old) were found only in the cord (total 8 thrombi in 6 cases). These findings probably relate to an acute event and indicate that cord’s thrombi may correlate with sudden fetal death.

Finally, a statistically significant temporal correlation between the onset of the thrombus inside the placental and fetal vessels and fetal death was not found, and a correlation between the presence of the thrombus and the death of the fetus was not demonstrated.

The analysis of the results obtained reveals the complexity of defining an appropriate method for the timing of thrombus age. This is mainly due to the lack of controlled experimental studies on the chronology of the thrombus in human tissues. Controlled studies on the chronology of skin lesions have been performed on humans under anesthesia [16]; however, ethical issues do not allow to design a human model of controlled thrombi formation.

Regarding human studies on the chronology of thrombus, the most considered are those of Irninger and Fineschi [9, 10]. Although they refer to timeframes too wide to be useful in TT-IUFD cases, they document the time of appearance of features like neovascularization and iron and calcium deposition. Therefore, starting from the observation in our cases that the presence of macrophages in the thrombus increases with the decrease of neutrophils, and this increase is accompanied by the appearance of hemosiderin + cells, calcium deposition, and angiogenesis, it has been possible to hypothesize the evolution of the thrombus from the first hours to the first days. By applying the Levenberg–Marquardt statistical test, taking as reference timing documented in literature (i.e., 4 days for hemosiderin + cells and angiogenesis, and < 7 days for calcium deposition), it has been possible to have a cellular kinetic curve that ranges from few hours to few days.

Our results suggest the possible usefulness of the N/M ratio for evaluating thrombus age and might be considered as a potential new parameter for timing estimation of very fresh thrombi in human tissues. The study must be confirmed through the application of the method also to other types of thrombus, such as, e.g., thrombus of the lower limbs and pulmonary thromboembolism.

## Conclusion

Our study confirms the maternal risk factors for fetal intrauterine death, and how the pathologies of the cord, followed by those of the placental parenchyma, are the conditions that are most frequently associated with the presence of thrombi. However, in our casuistry, there are still several cases in which no maternal risk factors are identified and the timing of the thrombus may be an additional element of support in pathology diagnostics and in medico-legal evaluations, especially in cases in which the death of the fetus takes place without known risk factors.

In legal medicine, the chronology of the first hours of any injury is requested by the Public Prosecutor, even if mostly difficult to investigate. In fact, the problem of the causal connection is very often linked to hours; therefore, studies using ranges of weeks are not always useful for judicial purposes.

The study presented suggests the N/M ratio as a parameter to be used, together with others, i.e., hemosiderophagi, calcium deposition, and angiogenesis, for thrombi’s age determination, particularly in the first 24 h.

## Supplementary Information

Below is the link to the electronic supplementary material.

Supplementary file1 (PDF 126 KB)

## Data Availability

Not applicable.
