# Supplementary material for: Third trimester intrauterine fetal death: proposal for the assessment of the chronology of umbilical cord and placental thrombosis
Source: Int J Legal Med. 2022 Feb 11;136(3):705–11. doi: 10.1007/s00414-022-02784-3 (PMC9005411; doi:10.1007/s00414-022-02784-3)
Supplement: Supplementary file 1 — Supplementary file1 (PDF 126 KB) [file 414_2022_2784_MOESM1_ESM.pdf]

Thrombus	Location	Morphology	Ratio N/M	CD31	CD61	Actin	Iron	Von Kossa	Picro Mallory	Timing
1	Cord (artery)	Intravascular subocclusive	28,1	-	+ (cluster)	-	-	-	+	1 d
2	Cord insertion	Intravascular subocclusive	20,1	-	+	-	-	-	+	1 d
3	Cord insertion	Intravascular subocclusive	18,9	-	+	-	-	-	+	1 d
4	Cord (vein)	Intravascular subocclusive	16,3	-	+	-	-	-	+	1 d
5	Chorial plate	Intravascular subocclusive	13,7	-	+ (cluster)	-	-	-	+ (strands)	1 d
6	Chorial plate	Murale Intravascular subocclusive	12,6	-	+ (cluster)	-	-	-	+	1 d
7	Cord (vein)	Intravascular subocclusive	11	-	+	-	-	-	+	1 d
8	Cord (vein)	Mural subocclusive	7,3	-	+	-	-	-	+	1 d
9	Cord (artery)	Endoluminale non occludente	7,1	-	+ (cluster)	-	-	-	+ (strands)	1 d
10	Cord insertion	Intravascular subocclusive	6,8	-	+	-	-	-	+	1 d
11	Cord (vein)	Mural intravascular subocclusive	6,5	-	+ (cluster)	-	-	-	+ (strands)	1 d
12	Chorial plate	Intravascular subocclusive	6,1	-	+	-	-	-	+ (strands)	1 d
13	Cord (vein)	Intravascular subocclusive	3,9	-	+	-	-	-	+	2-3 d
14	Cord insertion	Intravascular subocclusive	3,8	-	+	-	-	-	+	2-3 d
15	Cord (vein)	Mural intravascular subocclusive	2,5	-	+ (cluster)	-	-	-	+ (strands)	2-3 d
16	Cord insertion	Intravascular subocclusive	2,5	-	+	-	-	-	+	2-3 d
17	Chorial plate	Mural subocclusive	1,8	-	+	-	-	-	+	2-3 d
18	Chorial plate	Mural intravascular subocclusive	1,4	-	+	-	+	-	+	4-6d
19	Fetus brachiocephalic vein	Intravascular subocclusive	1,4	-	+	-	+	+	+	4-6 d

20	Cord insertion	Mural intravascular subocclusive	1,1	+	+ (cluster)	-	+	+	+	2-3 d
21	Chorial plate	Mural subocclusive	1	+	+ (cluster)	-	-	-	+ (strands)	4-6 d
22	Cord insertion	Transmural subocclusive	0,8	+	+	-	-	-	+ (strands)	4-6 d
23	Cord insertion	Intravascular subocclusive	0,7	+	+ (cluster)	-	+	-	+ (strands)	> 7 d
24	Cord insertion	Mural subocclusive	0,7	-	+	-	+	-	+	> 7 d
25	Chorial plate	Transmural subocclusive	0,7	-	+	-	-	-	+	> 7 d
26	Cord insertion	Intravascular subocclusive	0,7	+	+	-	-	-	+	> 7 d
27	Chorial plate	Mural intravascular occlusive	0,6	+	+	+	+	+	+	> 7 d
28	Cord insertion	Intravascular subocclusive	0,5	-	+	-	+	-	+	> 7 d
29	Cord insertion	Multiple, mural subocclusive	0,5	-	+	-	+	-	+	>7 d
30	Cord insertion	Mural subocclusive	0,4	-	+ (cluster)	-	+	-	+ (strands)	> 7 d
31	Fetus renal vein	Mural occlusive	0,4	+	+	+	+	+	+	> 7 d
32	Cord insertion	Mural subocclusive	0,3	-	+	+	-	+	+	> 7 d
33	Chorial plate	Mural intravascular subocclusive	0,3	-	+	-	+	-	+	> 7 d
34	Cord insertion	Mural subocclusive	0,2	-	+	+	+	+	+	> 7 d
35	Chorial plate	Mural subocclusive	0,2	-	+	+	-	-	+	>7 d
